# A Model to Predict the Expansion of *Trioza erytreae* Throughout the Iberian Peninsula Using a Pest Risk Analysis Approach

**DOI:** 10.3390/insects11090576

**Published:** 2020-08-27

**Authors:** Jacinto Benhadi-Marín, Alberto Fereres, José Alberto Pereira

**Affiliations:** 1 Centro de Investigação de Montanha (CIMO), ESA, Instituto Politécnico de Bragança, Campus de Santa Apolónia, 5300-253 Bragança, Portugal; jbenma@ipb.pt; 2 Instituto de Ciencias Agrarias, Consejo Superior de Investigaciones Científicas, ICA-CSIC, Calle Serrano 115 dpdo, 28006 Madrid, Spain; a.fereres@csic.es

**Keywords:** citrus, *Candidatus liberibacter*, huanglongbing, kernel model, spread, citrus greening disease

## Abstract

**Simple Summary:**

The African citrus psyllid *Trioza erytreae* is a natural vector of *Candidatus* *liberibacter* spp., the causal agent of the “citrus greening disease”. Citrus greening has not yet been detected in Europe; however, it represents a serious threat to citrus production. In this work, we parameterize a series of models to predict the expected spread of *T. erytreae* in the Iberian Peninsula since its introduction in 2014. Although *T. erytreae* was first detected in northwestern Spain, its detection and rapid spread around Porto (in Portugal) shortly afterward suggests a second entry point or transport of infested plant material from Spain. Among the developed models, the one that covered the known spread of *T. erytreae* best after 5 years was the kernel model with two simultaneous entry points. The invaded area predicted beyond the observed spread strongly suggests a physical and/or bioclimatic barrier preventing further spread of *T. erytreae*. Further development and refinement of models are crucial to accurately predicting the potential future spread of *T. erytreae* throughout the Iberian Peninsula. Accurate models will aid the development of successful management and regulatory programs.

**Abstract:**

Assessing the potential of spread of an introduced crop pest in a new country is crucial to anticipating its effects on crop production and deriving phytosanitary management toward reducing potential negative effects. Citrus production represents a key agricultural activity throughout the Mediterranean basin. The African citrus psyllid *Trioza erytreae* (del Guercio, 1918) (Hemiptera: Triozidae) is a natural vector of *Candidatus* *liberibacter* spp., the causal agent of the harmful disease huanglongbing (HLB) or “citrus greening disease”. In continental Europe, *T. erytreae* was detected for the first time in northwestern Spain in 2014. Pest risk analysis (PRA) approaches, such as modeling, consider both time and space components to predict the potential distribution of pests in a given region. In this work, we aim to parameterize a model able to predict the expected spread of *T. erytreae* in the Iberian Peninsula using three types of PRA models. The kernel model with two hypothetical entry points accurately predicted the distribution of *T. erytreae* with respect to latitude. This model should be further refined and validated to support decision-makers in the adoption of timely and successful management and regulatory measures against the spread of *T. erytreae* to other citrus-producing areas in Europe.

## 1. Introduction

Globalization has emerged as a result of income growth requirements by countries [1]. The intensification of world trade and people movements reflects an increase of transport networks that facilitate pathways for the introduction of species into new areas. Species introduced outside their natural range, either within a country or between countries, intentionally or unintentionally by human activities, are considered alien species [2]. If an alien species arrives in a favorable habitat (i.e., without environmental limitations, an abundance of resources, and low or lack of natural enemies), it may become an invasive pest. From an anthropocentric point of view, pests are organisms that contribute to a quantitative or qualitative reduction in crop yield, negatively affecting agriculture, desirable ornamentals, domestic animals, or the natural environment [3].

Worldwide, cultivated land ranges from 11% to 15% of the main land-use categories [4]. Globally, crop pests can lead to significant yield losses. For example, losses in production have been estimated of up to 29%, 31%, 37%, 40%, 50%, and 80% of soybean, maize, rice, potatoes, wheat, and cotton, respectively [5]. Moreover, different agricultural landscape traits, such as agricultural intensification, may result in increased pest damage [6]. Thus, once an alien pest species is detected within a new area, early pest risk analyses (PRAs) are crucial to assess its probability of spread. This helps us to understand and predict the magnitude of its impact (i.e., economic and social consequences) and derive subsequent management options for reducing the risk to an acceptable level through phytosanitary regulations [7]. In this context, the approach followed to do so should consider both time and space to determine the potential distribution of the pest [8,9].

*Citrus* spp. production in the Iberian Peninsula (lemons, limes, oranges, tangerines, mandarins, clementines, and satsumas) encompasses 315,309 ha of land [10]. The African citrus psyllid *Trioza erytreae* (del Guercio, 1918; Hemiptera: Triozidae) is a natural vector of the phloem-limited Gram-negative bacterium *Candidatus liberibacter* spp., the putative causal agent of huanglongbing (HLB) or “citrus greening disease” [11,12]. HLB is an incurable disease that caused losses of up to 74% in citrus production in Florida, USA [13]. *Trioza erytreae* was detected in the northwestern Iberian Peninsula in 2014 [14], although it was present in Madeira and the Canary Islands by 1994 and 2002, respectively [15]. The detection of *T. erytreae* raised the alarm among citrus stakeholders from Spain, Portugal, and other Mediterranean countries, which fear the subsequent spread of the pest and the possible emergence of the dangerous citrus greening disease [15]. To our knowledge, there has not been an intentional approach to assess the potential spread of *T. erytreae* throughout the Iberian Peninsula.

Robinet et al. (2012) [16] developed a pool of PRA models, allowing different case studies, each one according to the availability of information (e.g., economic, temporal, and spatial data), utilizing time and space as model dimensions and presence/absence or density as output variables: (1) Model A: Temporal spread over cells integrated with impact (LG-Econ) “logistic growth model based on economic values”, (2) Model B: Radial range expansion model (RR), (3) Model C: Population dynamics model (SLG) “Simple logistic growth model” or “temporal spread within cells”, and (4) Model D: dispersal kernel models (DK).

The objective of this work is to raise a model that is able to predict the geographical expansion of *T. erytreae* throughout the Iberian Peninsula after its accidental introduction using a generic spread model for pest risk analysis (PRA) under two scenarios: (1) a single entry point at the first detection site in northwest Spain and (2) two simultaneous entry points, the second one located at the nearest demographic hotspot in Porto (Portugal).

Among the tools provided by Robinet et al. (2012) [16] and according to the available information, three models were selected for parametrization: (1) a radial range expansion model (Model B, hereafter “radial model”), (2) a hybrid model of logistic growth and radial rate expansion (Models A and B, hereafter “random radial”), and (3) the deterministic version of the dispersal kernel model (hereafter “kernel model”). The kernel model can utilize various sets of parameters (see Robinet et al. (2012) in the supplementary material [16]). We used the one that is based on providing the known occurrence points of the species to predict future expansion.

## 2. Materials and Methods

### 2.1. Data Preparation

#### 2.1.1. Occurrence of *Trioza Erytreae* and Its Host, Citrus

The current occurrence of *T. erytreae* throughout the Iberian Peninsula was obtained from Spanish and Portuguese government technical reports [17,18,19] compiled for the period 2014–2019 and plotted on a digital elevation model from Reuter et al. (2007) [20]. Since *T. erytreae* feeds on several species of *Citrus* L., further analyses were conducted, considering the genus as the potential host. The occurrence records for *Citrus* spp. were obtained from the Global Biodiversity Information Facility online database [21].

#### 2.1.2. PRA Model Inputs

The suite of selected PRA models expects at least one variable indicating whether the species can potentially be established and another variable indicating how the population can grow. The bioclimatic suitability of *T. erytreae* and *Citrus* spp. were used as surrogates for each variable, respectively. Both variables were estimated using maxent entropy models. Maxent models are general-purpose machine learning methods that allow the modeling of species distribution using presence-only data [22].

The bioclimatic variables used as drivers were obtained from the WorldClim database [23] at 2.5 min spatial resolution (~4.5 km at the equator). A driver selection was carried out to avoid multicollinearity among the bioclimatic variables. Variables with a Pearson pairwise correlation >0.8 were excluded for modeling purposes. After an initial model performance assessment (see below), a second selection of bioclimatic variables was conducted, excluding those that did not contribute to predicting climatic suitability, according to their response curves.

The optimal model selection followed Muscarella et al. (2014) [24]. The “randomkfold” (kfolds = 2) method was used for *T. erytreae* with a combination of the linear, quadratic, product, and hinge feature classes and four regularization multipliers (β; 0.5, 1, 1.5, and 2), giving a total of 12 competing models. The “checkerboard1” method was used for *Citrus* spp. with five combinations of the linear, quadratic, product, threshold, and hinge feature classes and eight regularization multipliers (β; 0.5, 1, 1.5, 2, 2.5, 3, 3.5, and 4), giving a total of 40 competing models.

Each optimal model was assessed by selecting the one with the lowest AIC (Akaike information criterion) and was refitted using the optimal model tuning. Then, AUC (area under the curve), a threshold-independent measure of predictive accuracy based only on the ranking of locations [25], was calculated.

Finally, regarding the subsequent PRA models, the values of climatic suitability <0.1 and <0.25 for *T. erytreae* and *Citrus* spp., respectively, were cleaned to avoid an overestimation of the number of suitable cells throughout the Iberian Peninsula.

The maxent models were developed in R v.3.5.1 [26] using the R implementation of the maxent procedure [22].

### 2.2. Parameterization of PRA Models

The radial model requires an estimated radial rate of range expansion per year (RR; i.e., a rough estimate of potential spread) that has been calculated based on available data [14,27], from the introduction of the pest in August 2014 at Vilanova de Arousa Spain (42.562, −8.823) to the furthest record in November 2019 at Almada, Portugal (38.596, −9.168). This single parameter is enough to parameterize the radial model. The radial random model requires two additional parameters, the initial percentage of the risk area invaded at time *t* = 0 (N_0_), and the relative rate of spatial increase per year (r). N_0_ is estimated as

N_0_ = 100 × 1/N_max_

where N_0_ is the initial percentage of the risk area invaded at time *t* = 0, and N_max_ the number of suitable cells in the area.

The relative rate of spatial increase per year (r) is estimated using the linearization of the logistic equation for the number of invaded cells [16]:

Ln(N_t_/(N_max_ – N_t_)) – Ln[(N_0_/(N_max_ – N_0_)) + rt]

where N_0_ is the initial percentage of the risk area invaded at time *t* = 0, N_t_ is the number of invaded cells at time *t*, and N_max_ the number of suitable cells in the area.

The kernel model requires the population abundance at time *t* = 0, expressed as a percentage of the maximum population abundance (N_0_), the maximum year to year multiplication factor that a population can achieve under optimal conditions, assuming unlimited space (λ_max_), the proportion of the population engaged in dispersal (P), the shape parameter of the 2Dt dispersal kernel (ρ), and the scale parameter of the 2Dt dispersal kernel (u). Since no previous information on these parameters was available, P was assumed to be 1, following Robinet et al. (2012) [16] (i.e., all individuals are assumed to be engaged in dispersion), an initial estimate of u was approximated as u = RR [16]. N_0_, λ_max_, and ρ were estimated by calibration using a set of combinations among them (N_0_ = 0.00001, 0.000025, 0.00005, 0.0001; λ_max_ = 30, 40, 50; ρ = 3, 4, 5, 10, 15). A cell was considered invaded when population density, expressed as a percentage of the maximum abundance (carrying capacity), overcame 10^−6^.

Each model was run for five years, representing the potential evolution of spread from 2014 to 2019. For each simulation run, the predicted spread was visually compared to the known occurrence at *t* = 5 (year 2019). Since the hypothetic introduction point of *T. erytreae* into the Iberian Peninsula was Vilanova de Arousa, it was initially used to select the best model (i.e., among the three types of models) and to assess the performance of calibration of the kernel model. Finally, the best model was run again using the set of parameters that best fit the current known occurrence of *T. erytreae* (year 2019), considering the entry point at Vilanova de Arousa and a second hypothetical entry point located at Porto, Portugal (41.155, −8.612).

The PRA modeling process was conducted in R v.2.11.1 [28] using the R SpreadModule (v.8) [16] and a grid resolution of 10 km.

## 3. Results

### 3.1. Maxent Models for Citrus and Trioza erytreae

The retained explanatory variables as bioclimatic drivers (for descriptions see Table S1 online) for modeling the habitat suitability of *Citrus* spp., after the multicollinearity analysis, were mean diurnal range, isothermality, temperature seasonality, minimum temperature of the coldest month, annual range in temperature, mean temperature of the wettest quarter, mean temperature of driest quarter, precipitation of driest month, precipitation seasonality, and precipitation of the coldest quarter (see Figure S1 online).

Among the feature combinations and regularization multipliers used to select the optimal model (the one that achieved the lowest AIC) was the LQHP (linear, quadratic, hinge, product) model with a regularization multiplier β = 0.5, resulting in 111 parameters (see Table S2 online). The LQHP model gave AUC = 0.791 (see Figure S2 online). Among the selected bioclimatic variables, the drivers that contributed most to the maxent model were the precipitation seasonality (24.90%), temperature seasonality (21.20%), and mean temperature of the wettest quarter (19.50%) (see Table S3 online).

**Figure 1:**
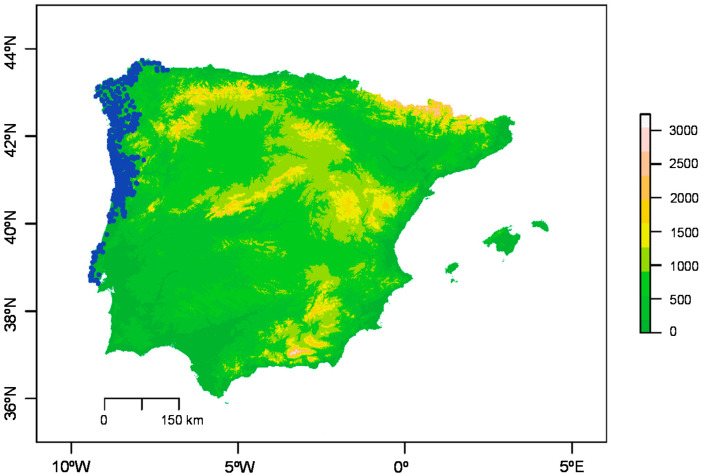
Occurrence-based distribution map of *Trioza erytreae* (del Guercio) throughout the Iberian Peninsula. Blue dots represent occurrence records projected on a digital elevation model. Altitude is expressed in meters (m). Results are shown for mainland Portugal and Spain and Balearic islands.

The occurrence of *T. erytreae* throughout the Iberian Peninsula is represented in Figure 1. For the estimation of the climatic suitability for *T. erytreae*, the only explanatory variable was precipitation in the coldest quarter (see Figure S3 online). The feature combination and regularization multiplier that achieved the lowest AIC was the LQHP model with a regularization multiplier β = 1, resulting in 6 parameters (see Table S4 online). The LQHP model gave AUC = 0.955 (see Figure S4 online). Since the only contributing variable was precipitation in the coldest quarter, the percent of contribution was 100% (see Table S5 online).

**Figure 2:**
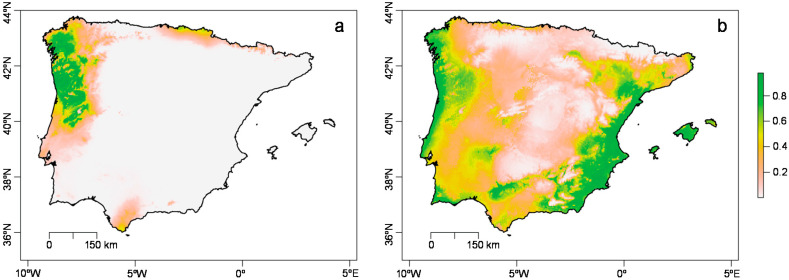
Maxent prediction model for climatic suitability of the huanglongbing vector *Trioza erytreae* (del Guercio) and its host (*Citrus* spp.) in the Iberian Peninsula. (**a**) *Trioza erytreae*. (**b**) *Citrus* spp. Colors indicate the gradient of bioclimatic suitability (p). Results are shown for mainland Portugal and Spain and Balearic islands.

The areas of best climatic suitability for *T. erytreae* and *Citrus* spp. were found to correspond to the northwestern area and the circumpeninsular area (excluding the northern area) of the Iberian Peninsula, respectively (Figure 2).

### 3.2. PRA Models

For all PRA models, the number of cells suitable for the establishment of *T. erytreae*, after filtering the climatic suitability provided by the *Citrus* spp. maxent model (i.e., retaining cells with *p* > 0.25), was 1534 (see Table S6 online). The radial model was parameterized with the radial rate of range expansion per year ≈130 km/year, giving 72.23% of niche invaded after a simulation of five years (see Table S6 and Figure S5 online). The radial random model was parameterized using the same radial rate of range of expansion per year, whereas the relative rate of spatial increase per year was estimated as *r* = 1.05, and the initial percentage of the risk area invaded at time *t* = 0 was estimated as N_0_ = 0.065, giving 11.08% of niche invaded after a simulation of five years (see Table S6 and Figure S6 online).

**Figure 3:**
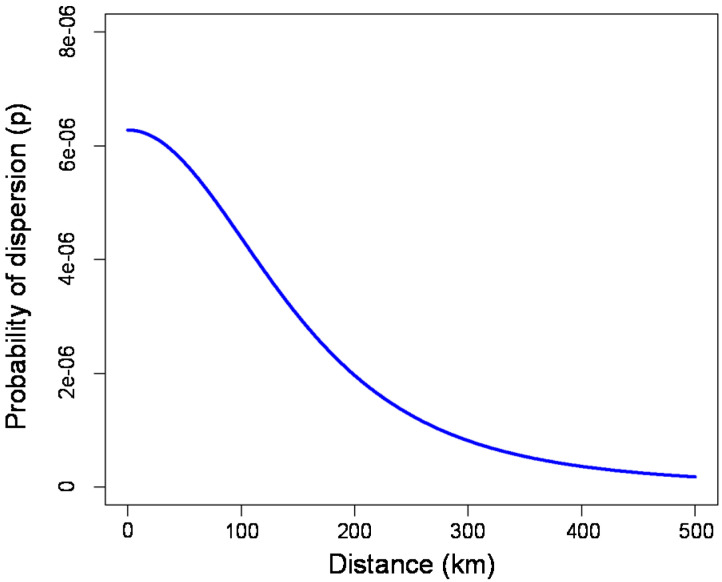
Dispersal kernel used to parameterize the kernel models after calibration (ρ = 3 and u = 130).

Among the 32 combinations of parameter values for the kernel models with a single entry point (see Table S6 and Figure S7 online), the model that best approximated the current occurrence of *T. erytreae* was the model parameterized with N_0_ = 0.0001, λmax = 30, ρ = 3, and u = 130 (Figure 3), giving 42.50% of niche invaded after a simulation of five years (see Table S6 and Figure S8 online).

**Figure 4:**
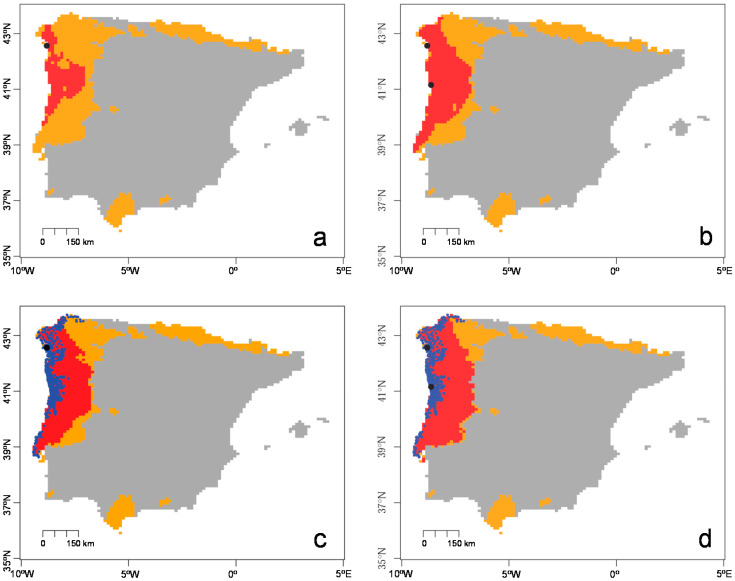
Predicted area invaded by *Trioza erytreae* using kernel models in a pest risk analysis framework. (**a**) Prediction for three years and one entry point. (**b**) Prediction for three years and two entry points. (**c**) Prediction for five years and one entry point. (**d**) Prediction for five years and two entry points. Orange areas represent favorable habitat suitability. Red areas represent invaded areas. Black dots represent entry points. Blue points represent the occurrence of *T. erytreae*. Results are shown for mainland Portugal and Spain and the Balearic islands.

**Figure 5:**
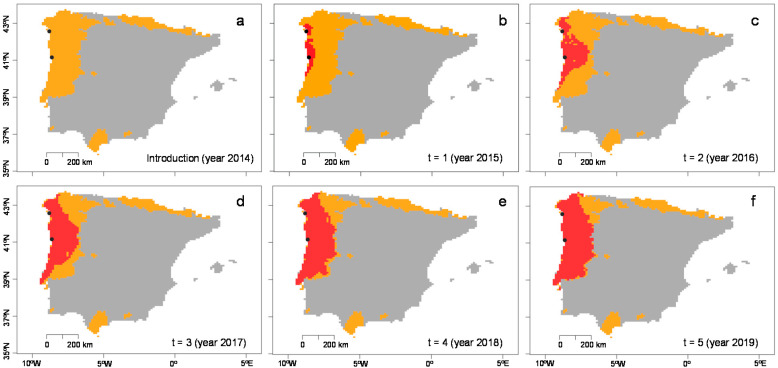
Evolution of the predicted area invaded by *Trioza erytreae* since its introduction in the Iberian Peninsula (**a**) using the kernel model with two entry points at Vilanova de Arousa (Spain) and Porto (Portugal) after one (**b**), two (**c**), three (**d**), four (**e**), and five years of simulation (**f**). Orange areas represent favorable habitat suitability. Red areas represent invaded areas. Black dots represent entry points. Results are shown for mainland Portugal and Spain and the Balearic islands.

The model that best represented the known spread of *T. erytreae* was the kernel model with two entry points (Figure 4), accurately covering the northernmost areas of Galicia (Spain) after five years (Figure 4d). With the same combination of parameters, the kernel model with two entry points gave 50.46% of niche invaded (see Figure 5 and Table S6 online).

## 4. Discussion

Several measures for preventing the introduction and spread of psyllid vectors and associated *Candidatus liberibacter* spp. in citrus include awareness, monitoring, surveillance, quarantine measures, and pest risk analysis. These steps are mandatory to protect the citrus industry in the Mediterranean region [29]. In the present work, distribution models (SDMs) for two species, *T. erytreae* and its preferred host, were developed using the maxent approach as inputs for dispersal models. This work contributes to the development of a tool that is able to predict and hopefully prevent the expansion of *T. erytreae*, currently the only vector of *Candidatus* *liberibacter* spp. throughout the Iberian Peninsula.

After the first detection of *T. erytreae* in the Iberian Peninsula in 2014, a series of delimiting surveys were carried out to assess its distributional range [30]. It was found that the population had spread from Vilanova de Arousa (Galicia, Spain) to the north, following the coastal line of Galicia, and to the south following the Portuguese coastal line, reaching the Lisbon region [31]. Currently, the known distribution of *T. erytreae* covers over 750 km of coastline from Galicia (Spain) to Almada (Portugal) (Figure 1) [17,18].

The geographic range of climatic suitability of *T. erytreae* (Figure 2a) predicted by the maxent model corresponds to its currently known coastal distribution but extends to inner mainland areas not currently colonized (~150 km to the east). The observed spread pattern since its introduction has been consistent with high values of bioclimatic suitability for *Citrus* spp. (Figure 2b), agreeing with the dependence of nymphal stages of *T. erytreae* on its host [22]. Using the available data, the only variable contributing to the bioclimatic suitability of *T. erytreae* in the Iberian Peninsula is precipitation in the coldest quarter of the year. The model predicted that the optimal climatic conditions for the occurrence of *T. erytreae* rely on precipitation ranging between 500 and 550 mm during the coldest quarter of the year (see Figure S3 online). This result will be relevant to future modeling approaches since spring outbreaks are driven by the size of overwintering populations of *T. erytreae* [32].

To our knowledge, this is the first species distribution model for *T. erytreae* in Europe. Our results agree to some extent with Richard et al. (2018) [33], who found that the precipitation in the wettest quarter was the strongest contributing driver to a maxent model developed for *T. erytreae* in Kenya. On the other hand, the potential bioclimatic suitability found for *T. erytreae* in the Iberian Peninsula using the maxent approach contrasts with other SDMs developed for the Asian citrus psyllid, *Diaphorina citri* Kuwayama (1908), the other main vector of citrus huanglongbing in the Americas and Asia. Narouei-Khandan et al. (2015) [34] found a near-null probability of occurrence of *D. citri* in the Iberian Peninsula using a maxent model, while a support vector machine model predicted high climate suitability for the Asian citrus psyllid on the southwestern and Mediterranean regions of the Iberian Peninsula, but not in the western part of the Iberian Peninsula. The latter distribution was also found by Gutierrez and Ponti (2013) [35] throughout the study area using an age-structured population dynamics model. Previous works on the climatic restrictions of *T. erytreae* have suggested that the combination of high temperatures and low relative humidity may strongly restrict the development of eggs and first instar nymphs [36]. This agrees with the current lack of both observed and predicted presence of *T. erytreae* in southern Portugal. Moreover, Green and Catling (1971) [37] found that the duration and seasonal distribution of near-lethal saturation deficit values could explain the geographic distribution of *T. erytreae*.

Regarding the PRA models, the radial and radial random models did not represent the observed pattern of expansion of the area invaded by *T. erytreae* during the five years after its introduction in the Iberian Peninsula (see Figures S5 and S6 online). This finding agrees with the circumpeninsular distribution of the host, regardless of the local scattered distribution of citrus crops and trees in small orchards and gardens within the study area [30].

Including dispersal in species distribution models is challenging because of the calibration of dispersal-related parameters such as the dispersal kernel or frequency of long-distance dispersal events [38]. Due to the high reproductive potential of *T. erytreae* under ideal conditions, considering a cell as “invaded” with a low density of individuals could be reasonable. Moreover, we found that the kernel model calibrated with a low value of the dispersal kernel was able to predict the latitudinal spread of *T. erytreae* from 2014 to 2019. A low dispersal kernel, between 1 and 5, according to Robinet et al. (2012) [16], represents a situation in which long-distance dispersal could be frequent. In this work, the use of ρ = 3 for the kernel model could reflect occasional spread events over long distances from anthropogenic causes such as traffic and the export of citrus fruits [16,30,39]. Furthermore, we cannot exclude long-distance transport of *T. erytreae* through air currents and low-level jet winds, as has been reported several times for insects of similar size such as aphids [40].

## 5. Conclusions

The pest risk analysis approach followed in this work was able to accurately predict the latitudinal spread of *T. erytreae* after five years using bioclimatic suitability for the pest and its host. This strongly suggests that the kernel model with two entry points deserves further research and refinement. For instance, incorporating geographical effects such as outbreaks and hot spots for local transport (see [30]), the effect of density-related patterns such as the preference of *T. erytreae* for crop borders [41], physical barriers (e.g., rivers, mountain chains), and high-resolution local climatic data would lead achieving a detailed and accurate tool to predict the future spread of *T. erytreae*. Although the Mediterranean basin is still free of HLB [42], the inefficient control measures against *T. erytreae* and its high rate of spread represent a significant threat, and this raises the alarm among scientists and stakeholders, especially regarding key citrus-growing areas in southern Portugal and southern and eastern Spain [19]. Thus, the development and refinement of both bioclimatic and spread models are critical to providing support to decision-makers toward the adoption of timely and successful management measures.

## Supplementary Materials

The following are available online at https://www.mdpi.com/2075-4450/11/9/576/s1. Figure S1: Response curve of each selected bioclimatic driver used to model the climatic suitability of *Citrus* in the Iberian Peninsula. Codes of variables correspond to Table S1. Figure S2: Receiver operating characteristic (ROC) curve of the maxent model developed for *Citrus*. Figure S3: Response curve of each selected bioclimatic driver used to model the climatic suitability of *Trioza eytreae* in the Iberian Peninsula. Codes of variables correspond to Table S1. Figure S4: Receiver operating characteristic (ROC) curve of the maxent model developed for *Trioza erytreae*. Figure S5: Predicted area invaded by *Trioza erytreae* using the radial model across five years (*t*) of simulation. Orange areas represent habitat suitability. Red areas represent invaded areas. Black dots represent entry points. Results are shown for mainland Portugal and Spain and the Balearic islands. Figure S6: Predicted area invaded by *Trioza erytreae* using the radial random model across five years (*t*) of simulation. Orange areas represent habitat suitability. Red areas represent invaded areas. Black dots represent entry points. Results are shown for mainland Portugal and Spain and the Balearic islands. Figure S7: Calibration of kernel model using a single entry point. Legends represent different combinations of N_0_, λ_max_, and ρ. a: N_0_ = 0.00001; b: N_0_ = 0.000025; c: N_0_ = 0.00005; d: N_0_ = 0.0001. Figure S8: Predicted area invaded by *Trioza erytreae* using the kernel model across five years (t) of simulation and a single entry point. Orange areas represent habitat suitability. Red areas represent invaded areas. Black dots represent entry points. Results are shown for mainland Portugal and Spain and Balearic islands. Table S1: Description of the WorldClim database bioclimatic variables. Table S2: Akaike information criterion (AIC) and number of parameters resulting in each combination among feature classes (FCs) and regularization multipliers (rms) during maxen model selection for *Citrus* spp. L: linear; Q: quadratic; P: product; T: threshold; H: hinge. Table S3: Percent of contribution of each retained bioclimatic driver used to model the climatic suitability of *Citrus* spp. in the Iberian Peninsula. Description of variable code in Table S1. Table S4: Akaike information criterion (AIC) and number of parameters resulting in each combination among feature classes (FCs) and regularization multipliers (rms) during maxen model selection for *Trioza erytreae*. L: linear; Q: quadratic; P: product; H: hinge. Table S5: Percent of contribution of the retained bioclimatic driver used to model the climatic suitability of *Trioza erytreae* in the Iberian Peninsula. Description of variable code in Table S1. Table S6: Parameters of each PRA model and results of parameterization by calibration for kernel models. *t*: years after introduction of *Trioza erytreae* in the Iberian Peninsula; riskcells: number of cells suitable for establishment of individuals; RR: radial rate of range expansion per year; u: scale parameter of the 2Dt dispersal kernel; r: relative rate of spatial increase per year; N_0_: initial percentage of the risk area invaded at time *t* = 0; lmax: maximum year to year multiplication factor (“finite growth rate”) that a population can achieve under optimal conditions, assuming unlimited space; p: shape parameter of the 2Dt dispersal kernel (number of degrees of freedom); Invaded: number of cells invaded at each year of simulation; pniche: percentage of niche invaded (riskcells) at each year of simulation. The proportion of the population engaged in dispersal (P) for kernel models was assumed to be 1 in all cases. Radial: radial model; RadialRand: Radial random model; kernel: kernel model with a single entry point at Vilanova de Arousa (Spain); kernel2: kernel model with an entry point at Vilanova de Arousa (Spain) and a second entry point at Porto (Portugal).
