# Supplementary material for: A Model to Predict the Expansion of Trioza erytreae Throughout the Iberian Peninsula Using a Pest Risk Analysis Approach
Source: Insects. 2020 Aug 27;11(9):576. doi: 10.3390/insects11090576 (PMC7563947; doi:10.3390/insects11090576)
Supplement: Supplementary file 1 [file insects-11-00576-s001.zip › insects-911057-supplementary.docx]

Supplementary

**Table S1.** Description of the WorldClim database bioclimatic variables.

| **Code** | **Description** |
| --- | --- |
| bio1 | Annual mean temperature |
| bio2 | Mean diurnal range (mean of monthly (max temp - min temp)) |
| bio3 | Isothermality (bio2/bio7) (× 100) |
| bio4 | Temperature seasonality (standard deviation × 100) |
| bio5 | Max temperature of warmest month |
| bio6 | Min temperature of coldest month |
| bio7 | Temperature annual range (bio5-bio6) |
| bio8 | Mean temperature of wettest quarter |
| bio9 | Mean temperature of driest quarter |
| bio10 | Mean temperature of warmest quarter |
| bio11 | Mean temperature of coldest quarter |
| bio12 | Annual precipitation |
| bio13 | Precipitation of wettest month |
| bio14 | Precipitation of driest month |
| bio15 | Precipitation seasonality (coefficient of variation) |
| bio16 | Precipitation of wettest quarter |
| bio17 | Precipitation of driest quarter |
| bio18 | Precipitation of warmest quarter |
| bio19 | Precipitation of coldest quarter |

**Table S2.** Akaike Information Criterion (AIC) and number of parameters resulting of each combination among feature classes (FC) and regularization multipliers (rm) during maxen model selection for *Citrus* spp.: L: linear; Q: quadratic; P: product; T: threshold; H: hinge.

| **Model** | **FC** | **rm** | **AIC** | **Parameters** |
| --- | --- | --- | --- | --- |
| 1 | L | 0.5 | 93928.12 | 9 |
| 2 | L | 1 | 94063.73 | 10 |
| 3 | L | 1.5 | 94172.93 | 8 |
| 4 | L | 2 | 94187.54 | 8 |
| 5 | L | 2.5 | 94207.70 | 8 |
| 6 | L | 3 | 94233.35 | 8 |
| 7 | L | 3.5 | 94264.02 | 8 |
| 8 | L | 4 | 94299.91 | 8 |
| 9 | LQ | 0.5 | 92304.04 | 17 |
| 10 | LQ | 1 | 92522.96 | 13 |
| 11 | LQ | 1.5 | 92657.29 | 13 |
| 12 | LQ | 2 | 92740.58 | 13 |
| 13 | LQ | 2.5 | 92854.69 | 12 |
| 14 | LQ | 3 | 92980.09 | 13 |
| 15 | LQ | 3.5 | 93086.90 | 13 |
| 16 | LQ | 4 | 93205.18 | 13 |
| 17 | LQH | 0.5 | 89378.62 | 108 |
| 18 | LQH | 1 | 89578.72 | 74 |
| 19 | LQH | 1.5 | 89807.34 | 72 |
| 20 | LQH | 2 | 90065.11 | 66 |
| 21 | LQH | 2.5 | 90234.33 | 47 |
| 22 | LQH | 3 | 90408.06 | 43 |
| 23 | LQH | 3.5 | 90598.15 | 42 |
| 24 | LQH | 4 | 90751.40 | 38 |
| 25 | LQHP | 0.5 | 89340.00 | 111 |
| 26 | LQHP | 1 | 89596.65 | 77 |
| 27 | LQHP | 1.5 | 89821.96 | 66 |
| 28 | LQHP | 2 | 90038.73 | 54 |
| 29 | LQHP | 2.5 | 90276.84 | 49 |
| 30 | LQHP | 3 | 90486.36 | 43 |
| 31 | LQHP | 3.5 | 90661.54 | 39 |
| 32 | LQHP | 4 | 90816.77 | 37 |
| 33 | LQHPT | 0.5 | 89347.87 | 247 |
| 34 | LQHPT | 1 | 89384.91 | 169 |
| 35 | LQHPT | 1.5 | 89605.67 | 137 |
| 36 | LQHPT | 2 | 89824.91 | 105 |
| 37 | LQHPT | 2.5 | 90018.29 | 87 |
| 38 | LQHPT | 3 | 90180.01 | 69 |
| 39 | LQHPT | 3.5 | 90339.51 | 65 |
| 40 | LQHPT | 4 | 90487.65 | 61 |

**Table S3.** Percent of contribution of each retained bioclimatic driver used to model the climatic suitability of *Citrus* spp. in the Iberian Peninsula. Description of variable code in Table S1.

| **Variable** | **Percent contribution (%)** |
| --- | --- |
| bio15 | 24.9 |
| bio4 | 21.2 |
| bio8 | 19.5 |
| bio6 | 13.6 |
| bio19 | 6.0 |
| bio3 | 5.5 |
| bio9 | 3.6 |
| bio14 | 3.2 |
| bio2 | 1.7 |
| bio7 | 0.8 |

**Table S4.** Akaike Information Criterion (AIC) and number of parameters resulting of each combination among feature classes (FC) and regularization multipliers (rm) during maxen model selection for *Trioza erytreae*. L: linear; Q: quadratic; P: product; H: hinge.

| **Model** | **FC** | **rm** | **AIC** | **Parameters** |
| --- | --- | --- | --- | --- |
| 1 | LQ | 0.5 | 11609.96 | 2 |
| 2 | LQ | 1 | 11620.45 | 2 |
| 3 | LQ | 1.5 | 11634.92 | 2 |
| 4 | LQ | 2 | 11651.93 | 2 |
| 5 | LQH | 0.5 | 11555.96 | 11 |
| 6 | LQH | 1 | 11565.90 | 11 |
| 7 | LQH | 1.5 | 11562.44 | 7 |
| 8 | LQH | 2 | 11569.33 | 8 |
| 9 | LQHP | 0.5 | 11559.20 | 12 |
| 10 | LQHP | 1 | 11554.01 | 6 |
| 11 | LQHP | 1.5 | 11559.74 | 6 |
| 12 | LQHP | 2 | 11564.30 | 7 |

**Table S5.** Percent of contribution of the retained bioclimatic driver used to model the climatic suitability of *Trioza erytreae* in the Iberian Peninsula. Description of variable code in Table S1.

| **Variable** | **Percent contribution (%)** |
| --- | --- |
| bio19 | 100 |

**Table S6.** Parameters of each PRA model and results of parameterization by calibration for kernel models. t: years after introduction of *Trioza erytreae* in the Iberian Peninsula; riskcells: number of cells suitable for establishment of individuals; RR: radial rate of range expansion per year; u: Scale parameter of the 2Dt dispersal kernel; r: relative rate of spatial increase per year; N0: Initial percentage of the risk area invaded at time t = 0; lmax: maximum year to year multiplication factor (“finite growth rate”) that a population could achieve under optimal conditions assuming unlimited space; p: shape parameter of the 2Dt dispersal kernel (number of degrees of freedom); Invaded: number of cells invaded at each year of simulation; pniche: percentage of niche invaded (riskcells) at each year of simulation. The proportion of the population engaged in dispersal (P) for kernel models was assumed to be 1 in all cases. Radial: radial model; RadialRand: Radial random model; kernel: kernel model with a single entry point at Vilanova de Arousa (Spain); kernel2: kernel model with an entry point at Vilanova de Arousa (Spain) and a second entry point at Porto (Portugal).

| **Model** | **t** | **riskcells** | **RR** | **u** | **r** | **N0** | **lmax** | **p** | **Invaded** | **pniche** |
| --- | --- | --- | --- | --- | --- | --- | --- | --- | --- | --- |
| Radial | 1 | 1534 | 130 | - | - | - | - | - | 292 | 19.0352 |
| Radial | 2 | 1534 | 130 | - | - | - | - | - | 679 | 44.26336 |
| Radial | 3 | 1534 | 130 | - | - | - | - | - | 929 | 60.56063 |
| Radial | 4 | 1534 | 130 | - | - | - | - | - | 994 | 64.79791 |
| Radial | 5 | 1534 | 130 | - | - | - | - | - | 1108 | 72.22947 |
| RadialRand | 1 | 1534 | 130 | - | 1.05 | 0.065 | - | - | 4 | 0.2607562 |
| RadialRand | 2 | 1534 | 130 | - | 1.05 | 0.065 | - | - | 9 | 0.5867014 |
| RadialRand | 3 | 1534 | 130 | - | 1.05 | 0.065 | - | - | 24 | 1.564537 |
| RadialRand | 4 | 1534 | 130 | - | 1.05 | 0.065 | - | - | 65 | 4.237288 |
| RadialRand | 5 | 1534 | 130 | - | 1.05 | 0.065 | - | - | 170 | 11.08214 |
| Kernel | 1 | 1534 | - | 130 | - | 0.00001 | 30 | 3 | 0 | 0 |
| Kernel | 2 | 1534 | - | 130 | - | 0.00001 | 30 | 3 | 0 | 0 |
| Kernel | 3 | 1534 | - | 130 | - | 0.00001 | 30 | 3 | 0 | 0 |
| Kernel | 4 | 1534 | - | 130 | - | 0.00001 | 30 | 3 | 0 | 0 |
| Kernel | 5 | 1534 | - | 130 | - | 0.00001 | 30 | 3 | 31 | 2.02086 |
| Kernel | 1 | 1534 | - | 130 | - | 0.00001 | 30 | 4 | 0 | 0 |
| Kernel | 2 | 1534 | - | 130 | - | 0.00001 | 30 | 4 | 0 | 0 |
| Kernel | 3 | 1534 | - | 130 | - | 0.00001 | 30 | 4 | 0 | 0 |
| Kernel | 4 | 1534 | - | 130 | - | 0.00001 | 30 | 4 | 16 | 1.043025 |
| Kernel | 5 | 1534 | - | 130 | - | 0.00001 | 30 | 4 | 127 | 8.279009 |
| Kernel | 1 | 1534 | - | 130 | - | 0.00001 | 30 | 5 | 0 | 0 |
| Kernel | 2 | 1534 | - | 130 | - | 0.00001 | 30 | 5 | 0 | 0 |
| Kernel | 3 | 1534 | - | 130 | - | 0.00001 | 30 | 5 | 0 | 0 |
| Kernel | 4 | 1534 | - | 130 | - | 0.00001 | 30 | 5 | 40 | 2.607562 |
| Kernel | 5 | 1534 | - | 130 | - | 0.00001 | 30 | 5 | 257 | 16.75359 |
| Kernel | 1 | 1534 | - | 130 | - | 0.00001 | 40 | 3 | 0 | 0 |
| Kernel | 2 | 1534 | - | 130 | - | 0.00001 | 40 | 3 | 0 | 0 |
| Kernel | 3 | 1534 | - | 130 | - | 0.00001 | 40 | 3 | 0 | 0 |
| Kernel | 4 | 1534 | - | 130 | - | 0.00001 | 40 | 3 | 42 | 2.73794 |
| Kernel | 5 | 1534 | - | 130 | - | 0.00001 | 40 | 3 | 264 | 17.20991 |
| Kernel | 1 | 1534 | - | 130 | - | 0.00001 | 40 | 4 | 0 | 0 |
| Kernel | 2 | 1534 | - | 130 | - | 0.00001 | 40 | 4 | 0 | 0 |
| Kernel | 3 | 1534 | - | 130 | - | 0.00001 | 40 | 4 | 5 | 0.3259452 |
| Kernel | 4 | 1534 | - | 130 | - | 0.00001 | 40 | 4 | 126 | 8.21382 |
| Kernel | 5 | 1534 | - | 130 | - | 0.00001 | 40 | 4 | 466 | 30.3781 |
| Kernel | 1 | 1534 | - | 130 | - | 0.00001 | 40 | 5 | 0 | 0 |
| Kernel | 2 | 1534 | - | 130 | - | 0.00001 | 40 | 5 | 0 | 0 |
| Kernel | 3 | 1534 | - | 130 | - | 0.00001 | 40 | 5 | 25 | 1.629726 |
| Kernel | 4 | 1534 | - | 130 | - | 0.00001 | 40 | 5 | 224 | 14.60235 |
| Kernel | 5 | 1534 | - | 130 | - | 0.00001 | 40 | 5 | 551 | 35.91917 |
| Kernel | 1 | 1534 | - | 130 | - | 0.00001 | 50 | 3 | 0 | 0 |
| Kernel | 2 | 1534 | - | 130 | - | 0.00001 | 50 | 3 | 0 | 0 |
| Kernel | 3 | 1534 | - | 130 | - | 0.00001 | 50 | 3 | 18 | 1.173403 |
| Kernel | 4 | 1534 | - | 130 | - | 0.00001 | 50 | 3 | 159 | 10.36506 |
| Kernel | 5 | 1534 | - | 130 | - | 0.00001 | 50 | 3 | 495 | 32.26858 |
| Kernel | 1 | 1534 | - | 130 | - | 0.00001 | 50 | 4 | 0 | 0 |
| Kernel | 2 | 1534 | - | 130 | - | 0.00001 | 50 | 4 | 0 | 0 |
| Kernel | 3 | 1534 | - | 130 | - | 0.00001 | 50 | 4 | 45 | 2.933507 |
| Kernel | 4 | 1534 | - | 130 | - | 0.00001 | 50 | 4 | 331 | 21.57757 |
| Kernel | 5 | 1534 | - | 130 | - | 0.00001 | 50 | 4 | 628 | 40.93872 |
| Kernel | 1 | 1534 | - | 130 | - | 0.00001 | 50 | 5 | 0 | 0 |
| Kernel | 2 | 1534 | - | 130 | - | 0.00001 | 50 | 5 | 0 | 0 |
| Kernel | 3 | 1534 | - | 130 | - | 0.00001 | 50 | 5 | 64 | 4.172099 |
| Kernel | 4 | 1534 | - | 130 | - | 0.00001 | 50 | 5 | 418 | 27.24902 |
| Kernel | 5 | 1534 | - | 130 | - | 0.00001 | 50 | 5 | 701 | 45.69752 |
| Kernel | 1 | 1534 | - | 130 | - | 0.000025 | 30 | 5 | 0 | 0 |
| Kernel | 2 | 1534 | - | 130 | - | 0.000025 | 30 | 5 | 0 | 0 |
| Kernel | 3 | 1534 | - | 130 | - | 0.000025 | 30 | 5 | 41 | 2.672751 |
| Kernel | 4 | 1534 | - | 130 | - | 0.000025 | 30 | 5 | 277 | 18.05737 |
| Kernel | 5 | 1534 | - | 130 | - | 0.000025 | 30 | 5 | 554 | 36.11473 |
| Kernel | 1 | 1534 | - | 130 | - | 0.000025 | 40 | 5 | 0 | 0 |
| Kernel | 2 | 1534 | - | 130 | - | 0.000025 | 40 | 5 | 13 | 0.8474576 |
| Kernel | 3 | 1534 | - | 130 | - | 0.000025 | 40 | 5 | 161 | 10.49544 |
| Kernel | 4 | 1534 | - | 130 | - | 0.000025 | 40 | 5 | 514 | 33.50717 |
| Kernel | 5 | 1534 | - | 130 | - | 0.000025 | 40 | 5 | 737 | 48.04433 |
| Kernel | 1 | 1534 | - | 130 | - | 0.000025 | 40 | 15 | 0 | 0 |
| Kernel | 2 | 1534 | - | 130 | - | 0.000025 | 40 | 15 | 44 | 2.868318 |
| Kernel | 3 | 1534 | - | 130 | - | 0.000025 | 40 | 15 | 333 | 21.70795 |
| Kernel | 4 | 1534 | - | 130 | - | 0.000025 | 40 | 15 | 647 | 42.17731 |
| Kernel | 5 | 1534 | - | 130 | - | 0.000025 | 40 | 15 | 817 | 53.25945 |
| Kernel | 1 | 1534 | - | 130 | - | 0.00005 | 30 | 2 | 0 | 0 |
| Kernel | 2 | 1534 | - | 130 | - | 0.00005 | 30 | 2 | 0 | 0 |
| Kernel | 3 | 1534 | - | 130 | - | 0.00005 | 30 | 2 | 0 | 0 |
| Kernel | 4 | 1534 | - | 130 | - | 0.00005 | 30 | 2 | 8 | 0.5215124 |
| Kernel | 5 | 1534 | - | 130 | - | 0.00005 | 30 | 2 | 40 | 2.607562 |
| Kernel | 1 | 1534 | - | 130 | - | 0.00005 | 30 | 3 | 0 | 0 |
| Kernel | 2 | 1534 | - | 130 | - | 0.00005 | 30 | 3 | 1 | 0.06518905 |
| Kernel | 3 | 1534 | - | 130 | - | 0.00005 | 30 | 3 | 58 | 3.780965 |
| Kernel | 4 | 1534 | - | 130 | - | 0.00005 | 30 | 3 | 248 | 16.16688 |
| Kernel | 5 | 1534 | - | 130 | - | 0.00005 | 30 | 3 | 485 | 31.61669 |
| Kernel | 1 | 1534 | - | 130 | - | 0.00005 | 30 | 4 | 0 | 0 |
| Kernel | 2 | 1534 | - | 130 | - | 0.00005 | 30 | 4 | 17 | 1.108214 |
| Kernel | 3 | 1534 | - | 130 | - | 0.00005 | 30 | 4 | 127 | 8.279009 |
| Kernel | 4 | 1534 | - | 130 | - | 0.00005 | 30 | 4 | 424 | 27.64016 |
| Kernel | 5 | 1534 | - | 130 | - | 0.00005 | 30 | 4 | 633 | 41.26467 |
| Kernel | 1 | 1534 | - | 130 | - | 0.00005 | 30 | 5 | 0 | 0 |
| Kernel | 2 | 1534 | - | 130 | - | 0.00005 | 30 | 5 | 30 | 1.955671 |
| Kernel | 3 | 1534 | - | 130 | - | 0.00005 | 30 | 5 | 209 | 13.62451 |
| Kernel | 4 | 1534 | - | 130 | - | 0.00005 | 30 | 5 | 507 | 33.05085 |
| Kernel | 5 | 1534 | - | 130 | - | 0.00005 | 30 | 5 | 711 | 46.34941 |
| Kernel | 1 | 1534 | - | 130 | - | 0.00005 | 30 | 10 | 1 | 0.06518905 |
| Kernel | 2 | 1534 | - | 130 | - | 0.00005 | 30 | 10 | 58 | 3.780965 |
| Kernel | 3 | 1534 | - | 130 | - | 0.00005 | 30 | 10 | 343 | 22.35984 |
| Kernel | 4 | 1534 | - | 130 | - | 0.00005 | 30 | 10 | 609 | 39.70013 |
| Kernel | 5 | 1534 | - | 130 | - | 0.00005 | 30 | 10 | 775 | 50.52151 |
| Kernel | 1 | 1534 | - | 130 | - | 0.00005 | 40 | 2 | 0 | 0 |
| Kernel | 2 | 1534 | - | 130 | - | 0.00005 | 40 | 2 | 0 | 0 |
| Kernel | 3 | 1534 | - | 130 | - | 0.00005 | 40 | 2 | 33 | 2.151239 |
| Kernel | 4 | 1534 | - | 130 | - | 0.00005 | 40 | 2 | 93 | 6.062581 |
| Kernel | 5 | 1534 | - | 130 | - | 0.00005 | 40 | 2 | 298 | 19.42634 |
| Kernel | 1 | 1534 | - | 130 | - | 0.00005 | 40 | 3 | 1 | 0.06518905 |
| Kernel | 2 | 1534 | - | 130 | - | 0.00005 | 40 | 3 | 43 | 2.803129 |
| Kernel | 3 | 1534 | - | 130 | - | 0.00005 | 40 | 3 | 210 | 13.6897 |
| Kernel | 4 | 1534 | - | 130 | - | 0.00005 | 40 | 3 | 499 | 32.52934 |
| Kernel | 5 | 1534 | - | 130 | - | 0.00005 | 40 | 3 | 697 | 45.43677 |
| Kernel | 1 | 1534 | - | 130 | - | 0.00005 | 40 | 5 | 4 | 0.2607562 |
| Kernel | 2 | 1534 | - | 130 | - | 0.00005 | 40 | 5 | 71 | 4.628422 |
| Kernel | 3 | 1534 | - | 130 | - | 0.00005 | 40 | 5 | 394 | 25.68449 |
| Kernel | 4 | 1534 | - | 130 | - | 0.00005 | 40 | 5 | 668 | 43.54628 |
| Kernel | 5 | 1534 | - | 130 | - | 0.00005 | 40 | 5 | 801 | 52.21643 |
| Kernel | 1 | 1534 | - | 130 | - | 0.00005 | 40 | 10 | 13 | 0.8474576 |
| Kernel | 2 | 1534 | - | 130 | - | 0.00005 | 40 | 10 | 134 | 8.735332 |
| Kernel | 3 | 1534 | - | 130 | - | 0.00005 | 40 | 10 | 499 | 32.52934 |
| Kernel | 4 | 1534 | - | 130 | - | 0.00005 | 40 | 10 | 747 | 48.69622 |
| Kernel | 5 | 1534 | - | 130 | - | 0.00005 | 40 | 10 | 913 | 59.5176 |
| Kernel | 1 | 1534 | - | 130 | - | 0.0001 | 30 | 2 | 1 | 0.06518905 |
| Kernel | 2 | 1534 | - | 130 | - | 0.0001 | 30 | 2 | 4 | 0.2607562 |
| Kernel | 3 | 1534 | - | 130 | - | 0.0001 | 30 | 2 | 35 | 2.281617 |
| Kernel | 4 | 1534 | - | 130 | - | 0.0001 | 30 | 2 | 68 | 4.432855 |
| Kernel | 5 | 1534 | - | 130 | - | 0.0001 | 30 | 2 | 186 | 12.12516 |
| Kernel | 1 | 1534 | - | 130 | - | 0.0001 | 30 | 3 | 20 | 1.3038 |
| Kernel | 2 | 1534 | - | 130 | - | 0.0001 | 30 | 3 | 59 | 3.846154 |
| Kernel | 3 | 1534 | - | 130 | - | 0.0001 | 30 | 3 | 261 | 17.01434 |
| Kernel | 4 | 1534 | - | 130 | - | 0.0001 | 30 | 3 | 488 | 31.81226 |
| Kernel | 5 | 1534 | - | 130 | - | 0.0001 | 30 | 3 | 652 | 42.50326 |
| Kernel | 1 | 1534 | - | 130 | - | 0.0001 | 30 | 4 | 31 | 2.02086 |
| Kernel | 2 | 1534 | - | 130 | - | 0.0001 | 30 | 4 | 104 | 6.779661 |
| Kernel | 3 | 1534 | - | 130 | - | 0.0001 | 30 | 4 | 375 | 24.44589 |
| Kernel | 4 | 1534 | - | 130 | - | 0.0001 | 30 | 4 | 601 | 39.17862 |
| Kernel | 5 | 1534 | - | 130 | - | 0.0001 | 30 | 4 | 755 | 49.21773 |
| Kernel | 1 | 1534 | - | 130 | - | 0.0001 | 30 | 5 | 37 | 2.411995 |
| Kernel | 2 | 1534 | - | 130 | - | 0.0001 | 30 | 5 | 142 | 9.256845 |
| Kernel | 3 | 1534 | - | 130 | - | 0.0001 | 30 | 5 | 441 | 28.74837 |
| Kernel | 4 | 1534 | - | 130 | - | 0.0001 | 30 | 5 | 670 | 43.67666 |
| Kernel | 5 | 1534 | - | 130 | - | 0.0001 | 30 | 5 | 785 | 51.1734 |
| Kernel | 1 | 1534 | - | 130 | - | 0.0001 | 40 | 3 | 42 | 2.73794 |
| Kernel | 2 | 1534 | - | 130 | - | 0.0001 | 40 | 3 | 147 | 9.58279 |
| Kernel | 3 | 1534 | - | 130 | - | 0.0001 | 40 | 3 | 440 | 28.68318 |
| Kernel | 4 | 1534 | - | 130 | - | 0.0001 | 40 | 3 | 653 | 42.56845 |
| Kernel | 5 | 1534 | - | 130 | - | 0.0001 | 40 | 3 | 770 | 50.19557 |
| Kernel | 1 | 1534 | - | 130 | - | 0.0001 | 40 | 4 | 50 | 3.259452 |
| Kernel | 2 | 1534 | - | 130 | - | 0.0001 | 40 | 4 | 244 | 15.90613 |
| Kernel | 3 | 1534 | - | 130 | - | 0.0001 | 40 | 4 | 540 | 35.20209 |
| Kernel | 4 | 1534 | - | 130 | - | 0.0001 | 40 | 4 | 745 | 48.56584 |
| Kernel | 5 | 1534 | - | 130 | - | 0.0001 | 40 | 4 | 874 | 56.97523 |
| Kernel | 1 | 1534 | - | 130 | - | 0.0001 | 40 | 5 | 57 | 3.715776 |
| Kernel | 2 | 1534 | - | 130 | - | 0.0001 | 40 | 5 | 287 | 18.70926 |
| Kernel | 3 | 1534 | - | 130 | - | 0.0001 | 40 | 5 | 575 | 37.4837 |
| Kernel | 4 | 1534 | - | 130 | - | 0.0001 | 40 | 5 | 761 | 49.60887 |
| Kernel | 5 | 1534 | - | 130 | - | 0.0001 | 40 | 5 | 920 | 59.97392 |
| Kernel | 1 | 1534 | - | 130 | - | 0.0001 | 50 | 3 | 55 | 3.585398 |
| Kernel | 2 | 1534 | - | 130 | - | 0.0001 | 50 | 3 | 263 | 17.14472 |
| Kernel | 3 | 1534 | - | 130 | - | 0.0001 | 50 | 3 | 547 | 35.65841 |
| Kernel | 4 | 1534 | - | 130 | - | 0.0001 | 50 | 3 | 745 | 48.56584 |
| Kernel | 5 | 1534 | - | 130 | - | 0.0001 | 50 | 3 | 874 | 56.97523 |
| Kernel | 1 | 1534 | - | 130 | - | 0.0001 | 50 | 4 | 68 | 4.432855 |
| Kernel | 2 | 1534 | - | 130 | - | 0.0001 | 50 | 4 | 330 | 21.51239 |
| Kernel | 3 | 1534 | - | 130 | - | 0.0001 | 50 | 4 | 614 | 40.02608 |
| Kernel | 4 | 1534 | - | 130 | - | 0.0001 | 50 | 4 | 781 | 50.91265 |
| Kernel | 5 | 1534 | - | 130 | - | 0.0001 | 50 | 4 | 936 | 61.01695 |
| Kernel | 1 | 1534 | - | 130 | - | 0.0001 | 50 | 5 | 76 | 4.954368 |
| Kernel | 2 | 1534 | - | 130 | - | 0.0001 | 50 | 5 | 364 | 23.72881 |
| Kernel | 3 | 1534 | - | 130 | - | 0.0001 | 50 | 5 | 659 | 42.95958 |
| Kernel | 4 | 1534 | - | 130 | - | 0.0001 | 50 | 5 | 819 | 53.38983 |
| Kernel | 5 | 1534 | - | 130 | - | 0.0001 | 50 | 5 | 944 | 61.53846 |
| Kernel2 | 1 | 1534 | - | 130 | - | 0.0001 | 30 | 3 | 89 | 5.801825 |
| Kernel2 | 2 | 1534 | - | 130 | - | 0.0001 | 30 | 3 | 315 | 20.53455 |
| Kernel2 | 3 | 1534 | - | 130 | - | 0.0001 | 30 | 3 | 531 | 34.61538 |
| Kernel2 | 4 | 1534 | - | 130 | - | 0.0001 | 30 | 3 | 699 | 45.56714 |
| Kernel2 | 5 | 1534 | - | 130 | - | 0.0001 | 30 | 3 | 774 | 50.45632 |

**Figure S1.** Response curve of each selected bioclimatic driver used to model the climatic suitability of *Citrus* in the Iberian Peninsula. Codes of variables correspond to Table S1.

**Figure S2.** Receiver operating characteristic (ROC) curve of the maxent model developed for *Citrus*.

**.**

**Figure S3.** Response curve of each selected bioclimatic driver used to model the climatic suitability of *Trioza eytreae* in the Iberian Peninsula. Codes of variables correspond to Table S1.

**Figure S4.** Receiver operating characteristic (ROC) curve of the maxent model developed for *Trioza erytreae*.

**Figure S5.** Predicted area invaded by *Trioza erytreae* using the radial model across five years (t) of simulation. Orange areas represent habitat suitability. Red areas represent invaded areas. Black dots represent entry points. Results are shown for mainland Portugal and Spain and Balearic islands.

**Figure S6.** Predicted area invaded by *Trioza erytreae* using the radial random model across five years (t) of simulation. Orange areas represent habitat suitability. Red areas represent invaded areas. Black dots represent entry points. Results are shown for mainland Portugal and Spain and Balearic islands.

**Figure S7.** Calibration of kernel model using a single entry point. Legends represent different combination of N_0_, λ_max_, and ρ. a: N_0_ = 0.00001. b: N_0_ = 0.000025. c: N_0_ = 0.00005. d: N_0_ = 0.0001.

**Figure S8.** Predicted area invaded by *Trioza erytreae* using the kernel model across five years (t) of simulation and a single entry point. Orange areas represent habitat suitability. Red areas represent invaded areas. Black dots represent entry points. Results are shown for mainland Portugal and Spain and Balearic islands.
